# Investigation on the characteristics and mechanisms of ACE inhibitory peptides by a thorough analysis of all 8000 tripeptides via binding free energy calculation

**DOI:** 10.1002/fsn3.2253

**Published:** 2021-05-03

**Authors:** Ruiyao Chen, Yulu Miao, Xuan Hao, Bei Gao, Mingzhe Ma, John Z.H. Zhang, Rui Wang, Sha Li, Xiao He, Lujia Zhang

**Affiliations:** ^1^ State Key Laboratory of Bioreactor Engineering School of Biotechnology East China University of Science and Technology Shanghai China; ^2^ Shanghai Engineering Research Center of Molecular Therapeutics and New Drug Development School of Chemistry and Molecular Engineering East China Normal University Shanghai China; ^3^ NYU‐ECNU Center for Computational Chemistry at NYU Shanghai Shanghai China; ^4^ Department of Chemistry New York University New York NY USA; ^5^ College of Food Science and Light Industry Nanjing Tech University Nanjing China; ^6^ State Key Laboratory of Materials‐Oriented Chemical Engineering Nanjing Tech University Nanjing China

**Keywords:** ACE inhibitor, in silico experiment, inhibitory mechanism, tripeptides

## Abstract

Food‐derived angiotensin I‐converting enzyme (ACE) inhibitory peptides represent a potential source of new antihypertensive. However, their characteristics and binding mechanisms were not well understood. In this study, novel energy calculation and experimentation were combined to elucidate the characteristics and mechanisms of ACE inhibitory tripeptides. ACE inhibitory activity of all 8,000 tripeptides was investigated by in silico experiments. IC_50_ values of the five top‐rated tripeptides ranged from 5.86 to 21.84 μM. Five hundred top‐ranked tripeptides were chosen for detailed structure–activity analysis, and a significant preference for aromatic amino acids at both C‐ and N‐terminus was found. By binding free energy analysis of nine representative tripeptides via MM/GBSA, electrostatic energy was found to be the leading energy that contributed to the binding of ACE with its high affinity tripeptides. Besides, S355, V380, and V518, three residues positioned around the classical binding pockets of ACE, also played a key role in ACE's binding. Therefore, for tripeptides, their binding pockets in ACE were redefined. In conclusion, the characteristics of ACE inhibitory peptides were more deeply illustrated by the thorough analysis of all tripeptides. The energy analysis allows a better understanding of the binding mechanisms of ACE inhibitory peptides, which could be used to redesign the ACE inhibitors for stronger inhibitory activity.

## Graphical abstract

The characteristics of C‐ and N‐terminus of ACE inhibitory tripeptides were investigated. Electrostatic energy contributed to the binding of ACE with its high affinity tripeptides.

S355, V380, and V518 played a key role ACE’s binding with tripeptides.

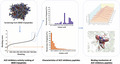

## 1 INTRODUCTION

Cardiovascular disease (CVD) will result in more than 23.6 million deaths each year by 2030 (Deshwal et al., 2017). Among the risk factors for CVD, high blood pressure (BP) is associated with the strongest evidence for causation and it has a high prevalence of exposure. BP is associated with different biochemical pathways. Renin–angiotensin system (RAS) and kallikrein–kinin system (KKS) are pivotal regulators that control BP. Within RAS, ACE catalyzes the conversion of inactive angiotensin I to angiotensin II, which is a powerful vasoconstrictor (Natesh et al., 2003). Within KKS, ACE metabolizes the vasodilator bradykinin (Fu et al., 2017). Accordingly, blood pressure can be lowered by the inhibition of ACE activity. Currently, ACE inhibitors, including captopril and lisinopril, have achieved widespread usage in clinical treatments of hypertension. However, they can cause undesirable adverse effects, such as hypotension, renal failure, and dysgeusia (Brown & Vaughan, 1998). Thus, ACE inhibitory peptides derived from food have drawn significant attention as they may act as safe alternatives to ACE drugs, as well as additives in functional foods (Deng et al., 2017). ACE inhibitory peptides have been identified from a wide range of food proteins, such as Antarctic krill (Ji et al., 2017), seaweed (Admassu et al., 2018), olive seeds (Esteve et al., 2015), green soybeans (Hanafi et al., 2018), and *Chlorella vulgaris* (Xie et al., 2018). Among them, short chain peptides like tripeptides are point of focus, as they are predicted to be easily absorbed into the blood circulatory system, and less affected by gastrointestinal digestion (Martin & Deussen, 2017; Yu et al., 2020). Based on structure–activity analysis, several characteristics of the identified ACE inhibitory peptides have been found. For example, a C‐terminal Pro is more preferred by ACE inhibitory peptide (Garcia Mora et al., 2014).

The conventional approach for identifying ACE inhibitory peptides involves the generation of peptides encrypted in food proteins by enzymatic hydrolysis, followed by the isolation of ACE inhibitory fractions from hydrolysates via chromatographic techniques and their identification by mass spectrometry (Saadi et al., 2015). However, experiments are time‐consuming and labor costing. Furthermore, the active peptides may not be totally harvested due to the incomplete hydrolysis and peptide loss. Limited numbers of ACE inhibitory peptides restrict their structure–activity relationship study. In the last few years, theoretical calculation methods have been employed to study ACE inhibitory peptides, especially in the identification of them. For example, in our previous work, the relationship between docking scores and experimental IC_50_ values of 113 ACE inhibitory peptides was investigated, which could be used to predict the IC_50_ values of a peptide without doing experiments (Wu et al., 2014). Panyayai et al., (2018) identified several potential ACE inhibitors by virtual screening for the ACE inhibitory activity of all 8,000 tripeptides, which has shown that theoretical calculation method is superior in the ACE‐inhibitor identification as screening inhibitors from 8,000 tripeptides can be hardly achieved by experimentations. However, despite an increasing number of ACE inhibitory peptide sequences have been identified, the structural features that govern the ACE inhibitory properties of peptides and the binding mechanisms of ACE with inhibitors are still not well understood. Therefore, in this study, energy analysis was employed to elucidate the characteristics and mechanisms of ACE inhibitory peptides. First, the ACE inhibitory activity of all 8,000 tripeptides was ranked by theoretical calculation methods. Five hundred top‐rated peptides were used for structure–activity relationship study. Furthermore, the inhibitory mechanisms of ACE inhibitory peptides were investigated by binding free energy analysis and free energy decomposition. The energy analysis of ACE/tripeptide complexes enhanced the comprehension of the mechanisms of ACE inhibitors.

## 2 MATERIALS AND METHODS

### 2.1 Chemicals

ACE from rabbit lung and hippuryl‐histidyl‐leucine (HHL) were sourced from Sigma Chemical Co. (St. Louis). Captopril, acetonitrile of HPLC grade, and trifluoroacetic acid (TFA) were obtained from Macklin Biochemical Co. All other reagents were of analytical grade.

### 2.2 Generation of tripeptide library

Python scripts were used to build the tripeptide library sequences, with 8,000 possible combinations generated. Three‐dimensional structures of the library were prepared with the Ligprep module in Maestro 2.9 (Schrödinger, LLC) and further minimized with OPLS3 force field parameters.

### 2.3 ACE preparation

The crystal structure of human ACE (PDB ID: 1O8A) was imported from the Protein Data Bank. Crystallographic water from the structure was removed, whereas hydrogen atoms were added and missing loops were rebuilt. The OPLS_2005 force field was selected for energy minimization. All steps were performed with the protein preparation wizard in Maestro.

### 2.4 Molecular docking of the tripeptide library with ACE

Docking simulations of the library were performed using Glide module in Maestro. The grid used for docking was created using standard parameters. The grid box coordinates were located at the Zn (II) position. A standard precision protocol was used for the ligand position screening.

### 2.5 MD simulation process

AMBER18 was used to perform MD simulations. The leap module and ff14SB force field were used for the accomplishment of missing atoms and obtaining of necessary parameters, respectively. Cationic dummy atom (CaDA) approach was employed to treat zinc (II) in ACE. The TIP3P water box with 10 Å buffer was used as the solvent environment, and sodium ions were added to the system to maintain neutral. Next, the steepest descent and conjugate gradient methods were applied for the energy minimization. The system was heated to 300 K with 10 kcal/(mol * Å^2^) restraint. Chemical bonds, including hydrogen atoms, were constrained using the SHAKE algorithm. MD simulation was performed for 50 ns.

## 3 MM/GBSA

The MM/GBSA method can be expressed by the following equation:

(1) $$\Delta G_{\text{bind}}=\Delta G_{\text{solv}}+\Delta G_{\text{gas}}$$

Where $\Delta G_{\text{solv}}$ and $\Delta G_{\text{gas}}$ represent solvation free energy and gas‐phase energy, respectively. $\Delta G_{\text{gas}}$ is calculated by the following equation:

(2) $$\Delta G_{\text{gas}}=\Delta E_{\text{vdW}}+\Delta E_{\text{ele}}$$

Where $\Delta E_{\text{ele}}$ and $\Delta E_{\text{vdW}}$ represent electrostatic interaction and van der Waals (vdW) interaction. $△{\text{G}}_{\text{solv}}$ contains polar and nonpolar solvation free energy as follows:

(3) $$\Delta G_{\text{solv}}=\Delta G_{\text{np}}+\Delta G_{\text{gb}}$$

The polar solvation free energy $\Delta G_{\text{gb}}$ is calculated by the GB module, while the nonpolar solvation free energy $\Delta G_{\text{np}}$ is calculated by:

(4) $$\Delta G_{\text{np}}=\gamma \times \text{SASA}\;+\beta$$

The *SASA* is the solvent‐accessible surface area and calculated by the MSMS program (Sanner et al., 1996). The β and γ are set to 0.00 kcal·mol^‐1^ and 0.005 kcal·(mol·Å^2^)^‐1^, respectively.

### 3.1 Peptide synthesis

Potential ACE inhibitory peptides selected based on the docking results were solid phase synthesized from Bankpeptide Biological Technology CO., Ltd. The purity (>95%) of these peptides was verified by RP‐HPLC on an Inertsil ODS‐SP column (4.6 mm × 250 mm, 5 μm, Shimadzu Co. Ltd.). Sequences were verified by mass spectrometry (Micromass ZQ, Waters).

### 3.2 Assay of ACE inhibitory activity

ACE inhibitory activity was measured in vitro, using the method reported by Cushman and Cheung (1971) with modifications. Briefly, 20 μL of inhibitory peptides at different concentrations and 20 μL of 5 mM HHL solution were preincubated at 37°C for 5 min in 90 μL of buffer A (50 mM HEPES, 300 mM NaCl, pH 8.3). Next, 20 μL of ACE solution (0.04 U/mL) was added to trigger the reaction. After incubation at 37°C for 1 hr, the reaction was terminated by adding 50 μL of 1 M HCl. Next, 10 μL of the mixture was separated through RP‐HPLC (e2679, Waters) using an XBridge C_18_ column (4.6 mm × 150 mm, 5 μm, Waters). The column was eluted by 25% acetonitrile in 0.1% trifluoroacetic acid (v/v) with a flow rate at 1 mL/min. Hippuric acid was monitored at 228 nm. Triplicate tests were performed for each sample. The IC_50_ value was defined as the concentration of the peptide required to reduce the hippuric acid peak area by 50%. For comparative purposes, the IC_50_ value of captopril was obtained using this methodology.

## 4 RESULTS AND DISCUSSION

### 4.1 Docking results of the tripeptide library

The combinations of 8,000 tripeptides were firstly generated by python scripts, and their 2D structures were converted to 3D structures. Then, 8,000 tripeptides were successfully docked into ACE, and the best pose of each peptide was chosen according to their docking scores. Next, the 8,000 docking scores were ranked. The complete ranking list is given in Table S1. A lower docking score indicates that the peptide in question is a potential ACE inhibitor, as it binds with ACE in a more favorable conformation.

To investigate the validity of the docking results, five top‐ranked peptides (WWW, KYY, WRF, WRY, and WQW) and two bottom‐ranked peptides (DGG and GGG) were synthesized, and their IC_50_ values were tested in vitro (Table 1). The five top‐ranked peptides were potential ACE inhibitors with IC_50_ values ranging from 5.86 to 21.84 μM. Though the ACE inhibitory activity of these peptides was weaker than that of captopril (IC_50_ = 0.037 ± 0.008 μM), it was reported that ACE inhibitory peptides could be healthier than ACE drugs (Deng et al., 2017). In contrast, the bottom‐ranked peptides DGG and GGG showed poor inhibitory activity with IC_50_ values higher than 5,000 μM. Therefore, the docking results obtained in this study are reliable and can be used further for ACE inhibitory peptide studies.

**TABLE 1:** IC_50_ values of the representative peptides

Ranking	Docking score	Sequence	IC_50_ (μM)
1	−11.012	WWW	7.30 ± 2.11
2	−10.777	KYY	20.46 ± 4.05
3	−10.616	WRF	21.84 ± 2.50
4	−10.613	WRY	5.86 ± 0.73
5	−10.603	WQW	11.83 ± 1.79
7,999	−1.778	DGG	>5,000
8,000	−1.604	GGG	>5,000
		Captopril	0.037 ± 0.008

Note

Data are expressed as mean ± *SD*.

**FIGURE 1:**
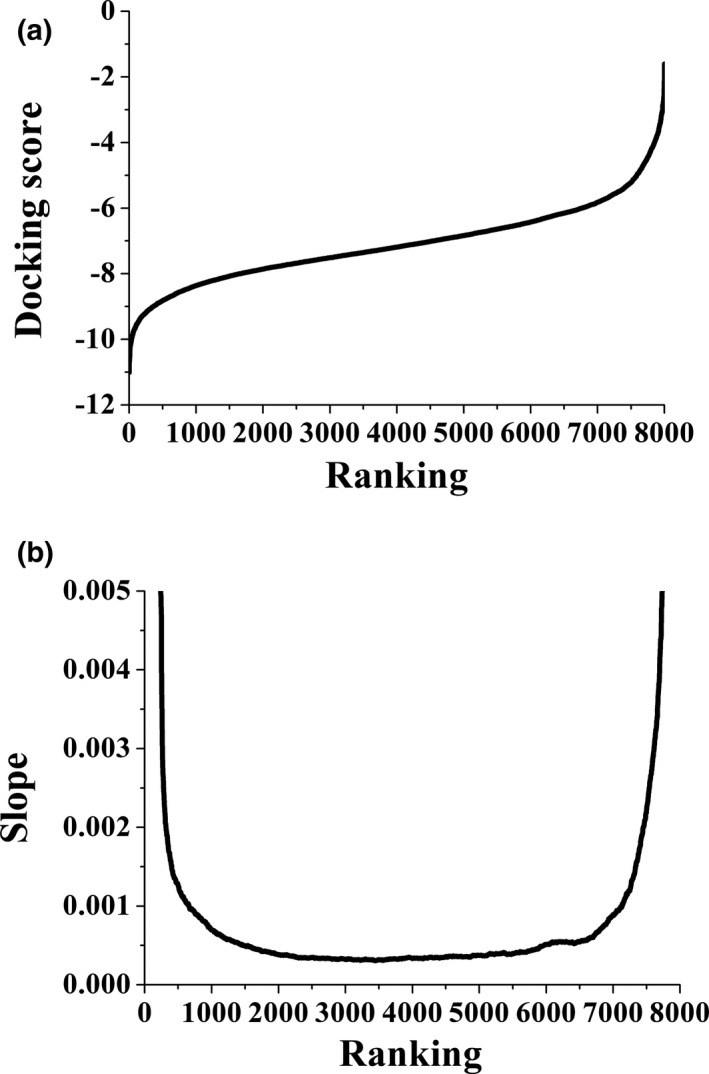
The docking score curve of the tripeptide library (a). The sloping curve of the docking score (b)

Figure 1a illustrates the distribution of 8,000 docking scores. Interestingly, the docking scores of top‐ranked and bottom‐ranked peptides increased quickly, whereas a huge proportion of peptides with medium scores showed a relatively slow increase in scores. Therefore, the slope of the curve in Figure 1a was analyzed. As is clearly shown in Figure 1b, the slopes of the peptides ranked before No.500 and after No. 7500 change dramatically compared with those of peptides ranked between No. 501 and No. 7499. The highest ranked peptide had a docking score of −11.012, and this value increased to −8.816 for the peptide ranked at No. 500. The docking score of the peptide ranked at No. 7500 was −5.218, whereas this value was −1.604 for the peptide ranked at No.8000. Although the 500 top‐ and bottom‐ranked peptides only accounted for 6.25% of the 8,000 peptides, respectively, their docking scores were in the 23.34% of the highest and 38.41% of the lowest within the score range, which indicated that only a small number of peptides are the most effective ACE inhibitors. Therefore, the 500 top‐ and bottom‐ranked peptides could represent the characteristics of ACE inhibitory and non‐ACE inhibitory peptides, which were good samples for structure–activity analysis.

### 4.2 The structure–activity analysis of ACE inhibitory peptides

**FIGURE 2:**
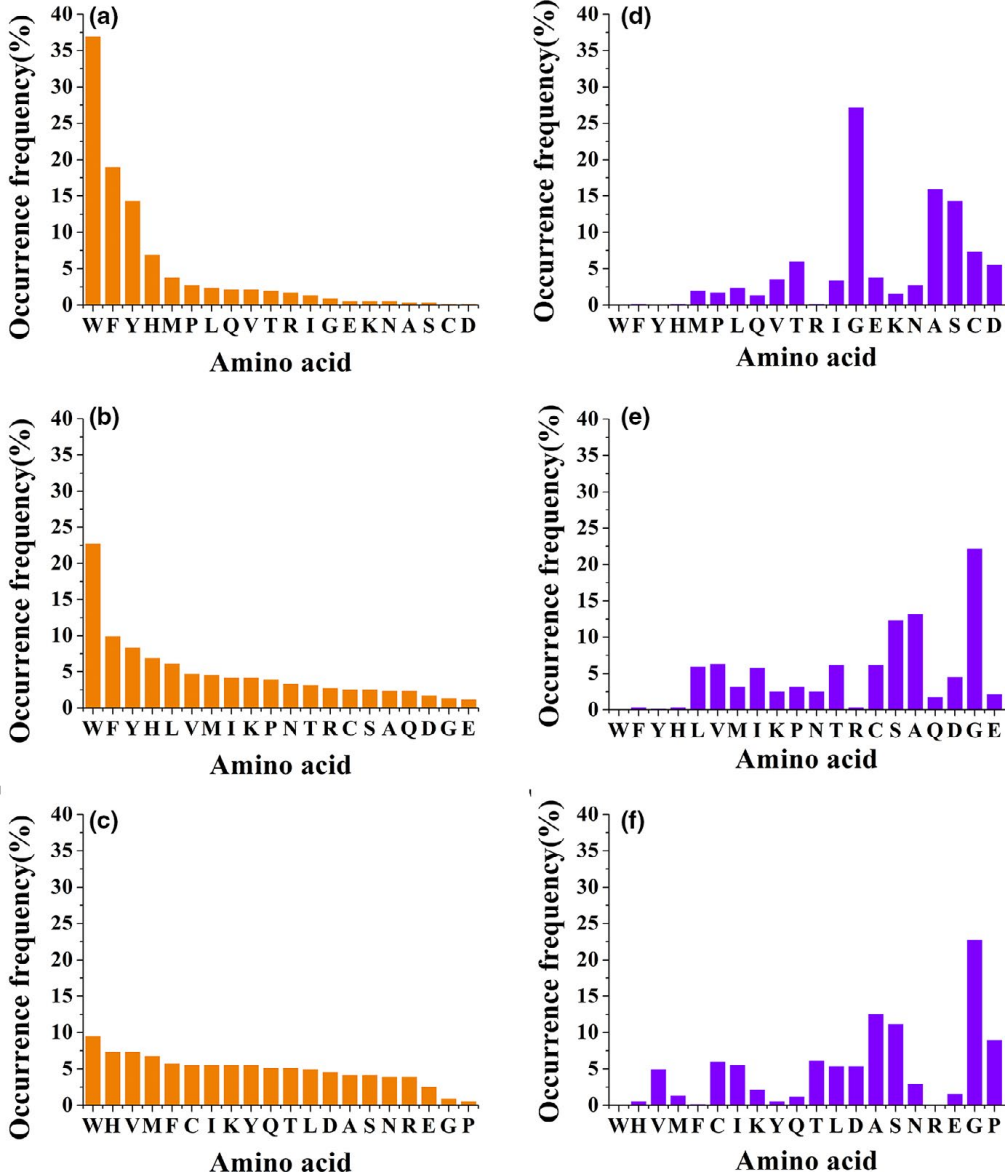
The occurrence frequency of 20 amino acids at the C‐terminus (a), N‐terminus (b), and middle position (c) within the 500 top‐ranked peptides and the C‐terminus (d), N‐terminus (e), and middle position (f) within the 500 bottom‐ranked peptides

Five hundred top‐ranked peptides were selected to investigate the occurrence frequency of 20 amino acids at the N‐, C‐, and internal positions. The results are illustrated in Figure 2a‐c. High‐frequency amino acids are preferential at particular positions.

As shown in Figure 2a, the occurrence frequency of 20 amino acids at the C‐terminus polarizes. W, F, and Y were the three most frequently occurring amino acids, with frequencies of 37.0%, 19.0%, and 14.4%, respectively. The total proportion of these three amino acids accounted for 70.4% of all amino acids, indicating that ACE inhibitory peptides strongly prefer C‐terminal aromatic amino acids. Similarly, a virtual screening for ACE inhibitory tripeptides also showed that aromatic amino acids were preferred at C‐terminus of the peptide (Panyayai et al., 2018). Several studies have previously isolated ACE inhibitory peptides with C‐terminal aromatic amino acids, such as IRW (Majumder & Wu, 2010), VAW (Rao et al., 2012), RGY (Rao et al., 2012), and IVF (Miguel et al., 2007). In addition, QSAR analysis showed that aromatic amino acids at the C‐terminus contribute to the ACE inhibitory activities of peptides (Wu et al., 2006). The occurrence frequency of peptides with His located at the C‐terminal position was 7%, which ranked fourth. Rodríguez‐Figueroa et al., (2012) identified DDQNPH from fermented milk and regarded the C‐terminal H as the main contributor to its potent ACE inhibitory activity. In addition, P is a commonly found amino acid at the C‐terminus in the highly active ACE inhibitory peptides (Orio et al., 2017). VAP (Chen et al., 2012) from grass carp and VWP and VNP (Chen et al., 2013) from rice possessed IC_50_ values of 18.6, 4.5, and 6.4 μM, respectively. IPP and VPP have both been applied for commercial use in Japan and Finland (Manikkam et al., 2016). In this study, the occurrence frequency of P was placed sixth among the 20 amino acids, demonstrating its significant role in ACE inhibitory peptides.

The four most frequently occurring amino acids for the N‐terminal of the ACE inhibitory peptide remained W, F, Y, and H, with frequencies of 22.8%, 10.0%, 8.4%, and 7.0%, respectively (Figure 2b). The high frequency of W, F, and Y indicated that peptides with N‐terminal aromatic amino acids were more likely to be ACE inhibitory peptides. This finding is in accordance with two previous studies (Garcia‐Redondo et al., 2010; Murray & FitzGerald, 2007). However, the total frequency of W, F, and Y at the C‐terminus was 70.4%, whereas the frequency at the N‐terminus was 41.2%. These frequencies indicate that the preference for N‐terminal aromatic amino acids is not as strong as for C‐terminal aromatic amino acids.

As shown in Figure 2c, no obvious preference for amino acid was observed in the middle positions. W remained the amino acid with the highest frequency. However, the subsequent ranking was no longer consistent with that of the C‐ and N‐terminus. The alkaline amino acid H, aliphatic amino acid V, and hydrophobic amino acid M ranked second, third, and fourth, respectively, and no obvious differences were found among the frequency of the top four amino acids (9.6%, 7.4%, 7.4%, and 6.8%). The frequency of the top‐ranked amino acid at the internal position was 1.7 times higher than that of the fifth‐ranked amino acid. In contrast, the ratio was 9.7 for the C‐terminus and 3.7 for the N‐terminus. This analysis revealed that the preference for a particular type of amino acid at the middle position was not as strong as that for the C‐ and N‐positions. The amino acid P had the lowest frequency at the middle position, indicating that an internal P may not be a good choice for ACE inhibitory tripeptides. This conclusion is consistent with a previous study by Miguel et al., (2012) wherein the C‐terminal P contributes to the ACE inhibitory activity, but the peptide with an internal position P binds weakly to ACE.

As shown in Figure 2d‐f, the 500 bottom‐ranked tripeptides possess opposite characteristics against the top‐ranked ones. Amino acids preferred by the top‐ranked peptides had low occurrence frequency in the 500 bottom‐ranked peptides. Among the bottom‐ranked tripeptides, G, A, and S were the top three most frequently occurring amino acids at N‐terminal, C‐terminal, and internal positions. This outcome suggested that peptides with G, A, and S may not be potent ACE inhibitory peptides. Interestingly, W did not appear in the 500 bottom‐ranked peptides either at the N‐terminal, C‐terminal, or internal positions, which also validated the contribution of W to the ACE inhibitory peptides.

The characteristics of ACE inhibitory peptides help us identify the potential antihypertensive foods. As mentioned above, ACE inhibitory tripeptides prefer C‐terminal W. Therefore, a W‐rich diet is more likely to reduce blood pressure. Chia seeds, edible seeds of chia plant, have a high concentration of W (440 mg/100 g) (Kałużna‐Czaplińska et al., 2019; Reuter et al., 2021). Some studies have shown a promising potential of chia seeds to inhibit the activity of ACE. The enzymatic hydrolysates of chia flour peptides possess ACE inhibitory activity with EC_50_ value of 516 μg/mL, which indicates that chia flour is a stronger ACE inhibitor than other seed flours (Orona‐Tamayo et al., 2015). Similarly, Chia seed oil industry meal by‐product can also be used as a source of bioactive peptide with antihypertensive potential (San Pablo‐Osorio et al., 2019). Furthermore, LW, NW, and YW, three peptides with C‐terminal W from chia seeds, have been identified as ACE inhibitors (Grancieri et al., 2019). In order to enhance the antihypertensive effects of chia seeds, specific proteases can be used to increase the content of useful peptides. For instance, proteinase K is suitable for the production of peptides with C‐terminal W, as they preferentially cleave after W (Saenger, 2013). After the second digestion by gastrointestinal enzymes, short peptides with high ACE activity are more likely to be obtained. The above methods are economical, safe, and effective antihypertensive strategies which can be applied in food industry.

### 4.3 Interactions of peptides with ACE

**FIGURE 3:**
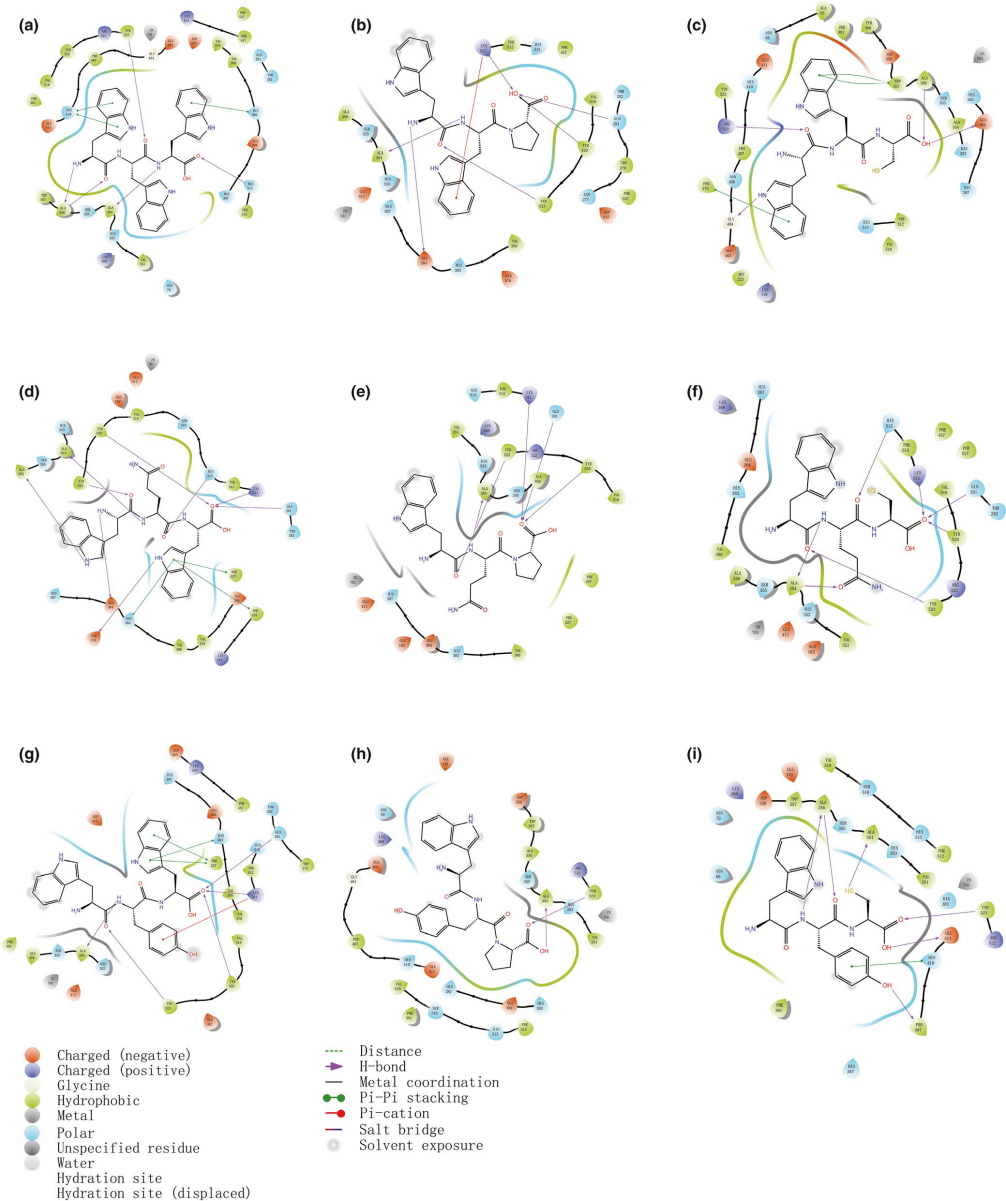
2d interaction of ACE with WWW (a), WWP (b), WWC (c), WQW (d), WQP (e), WQC (f), WYW (g), WYP (h), and WYC (i)

WWW, WQW, and WYW were the three top‐ranked peptides with C‐terminal W. In order to illustrate the binding mode of ACE inhibitors, the C‐terminal W of the three peptides was replaced by C and P, and the interactions between ACE and the nine peptides were investigated (Figure 3). ACE contains three binding pockets. A354, E384, and Y523 correspond to the S1 pocket, whereas Q281, H353, K511, H513, and Y520 correspond to the S2 pocket. E162 is the only amino acid contained in the S1’ pocket (Lan et al., 2015). H‐bond is the dominant interaction that stabilizes the ACE‐inhibitor complex (Xie et al., 2018; Yuan et al., 2018). Among the nine complexes, the interaction pattern of WWP/ACE, WYP/ACE, WQP/ACE, and WQC/ACE complexes was alike, as these peptides only form H‐bonds with residues in the binding pockets. WWC and WYC interacted with ACE in a different way. WWC only formed one H‐bond with S1 pocket residue E384, and three additional H‐bonds were formed with G404, R522, and A356. Similarly, as for WYC, two H‐bonds were formed with pocket residues A354 and Y523, and additional three H‐bonds were formed with A356, E411, and P407.

As well as H‐bonds, Zn(II) buried in the active sites is a vital catalysis component and it can coordinate with H383, H387, and E411 of ACE (Natesh et al., 2003). A docking simulation by Jimsheena and Gowda (2010) found that the antihypertensive drug lisinopril can coordinate with Zn(II) directly. However, no direct interactions of the nine studied tripeptides with Zn(II) were observed, which might explain their weaker inhibitory activities compared with lisinopril. Among the nine peptides, WWW, WYW, and WQW formed π‐π bonds with H383, and the π‐π bonds may interfere with the coordination effect of H383 with the Zn ion, which could enhance their inhibitory activities. Similar findings were reported by Abdelhedi et al., (2018) and Yu et al., (2018). Although the interaction analysis showed certain rules, further energy calculations were carried out to explore the binding mechanisms of ACE inhibitors.

### 4.4 Binding free energy analysis

**FIGURE 4:**
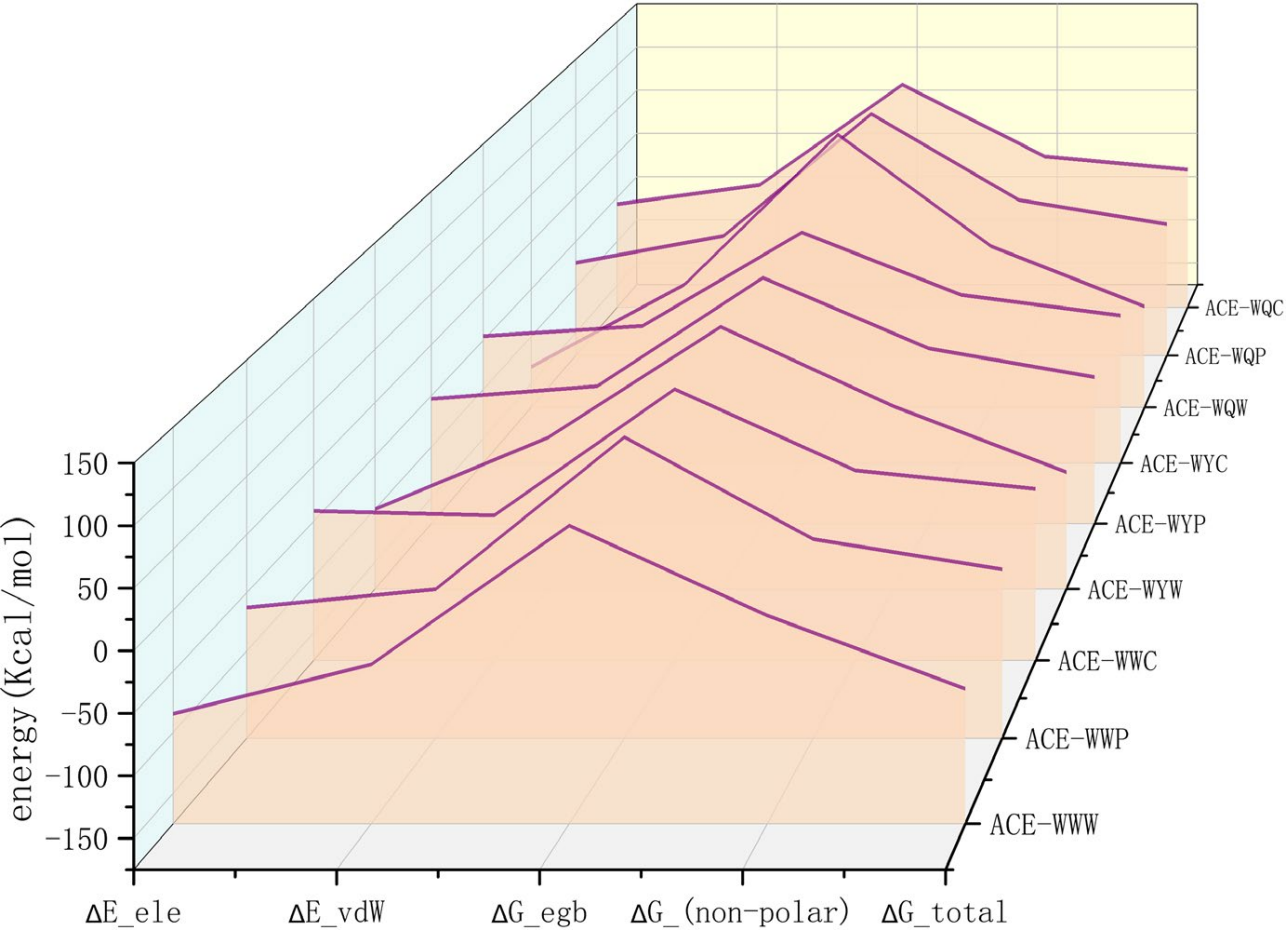
Binding free energies of ACE–peptides complexes obtained by MM/GBSA. $\Delta E_{\;\text{- ele}}$, $\Delta E_{\_\text{vdW}}$, $\Delta G_{\;\text{- eg}}$ and $\Delta G_{\_\text{non - polar}}$ are binding energy components of electrostatic, van der Waals, electrostatic solvation, and nonpolar solvation energies, respectively. $\Delta G_{\;\text{- total}}$ represents the total binding free energy

The binding free energies ($\Delta G_{\text{bind}}$) of the nine peptides with ACE were calculated by MM/GBSA (Figure 4). The binding free energies of peptides with C‐terminal W (WWW, WYW and WQW) were generally lower than those of peptides with C‐terminal P, while peptides with C‐terminal C (WWC, WYC, and WQC) possessed the highest $\Delta G_{\text{bind}}$ values among the nine peptides. A lower energy indicated a more stable complex. Therefore, the results for binding energy analysis were in accordance with the docking results, which further proved that C‐terminal W contributes to the binding of ACE inhibitors.

The binding free energy can be divided into four parts: van der Waals ($\Delta E_{\text{vdw}}$), electrostatic ($\Delta E_{\text{ele}}$), electrostatic solvation ($\Delta G_{\text{egb}}$), and nonpolar solvation energies ($\Delta G_{\text{non - polar}}$). Among the above energies, electrostatic energy of the nine complexes was quite different, which led to the diversity of the binding energy of the three types of peptide with ACE. For instance, electrostatic energy of ACE‐WWW was −84.856 Kcal/mol. When the C‐terminal W was replaced by P and C, $\Delta E_{\text{ele}}$ values decreased to −62.6586 Kcal/mol and −40.8025 Kcal/mol for ACE‐WWP and ACE‐WWC complexes, respectively. On the contrary, the $\Delta E_{\text{vdW}}$ and $\Delta G_{\text{non - polar}}$ values of the three types of peptides were very close. Interestingly, $\Delta G_{\text{egb}}$ value of all the nine complexes was positive, which indicated its negative role in the stability of the ACE–peptide complexes.

**FIGURE 5:**
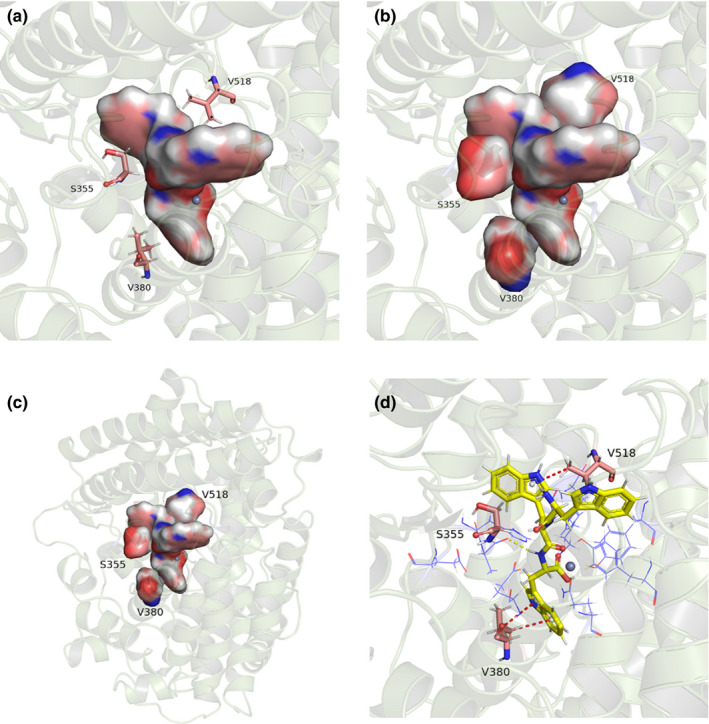
Positions of V518, S355, and V380 versus the classical binding pocket of ACE (a). Local (b) and general (c) overviews of the binding pocket of ACE containing Val518, Ser355, and Val380. The interactions between V518, S355, and V380 with WWW (d). Yellow dotted line indicates hydrogen bond formation. Red dotted lines indicate hydrophobic interactions. Residues from the classical binding pocket of ACE are shown in line model

To further gain insight into the binding mechanisms of peptide–ACE interaction, the contribution of every residue to the binding free energy of ACE/peptide complex was calculated by free energy decomposition and is shown in Table 2. Amino acids that contributed mostly to the binding of C‐terminal W and C‐terminal P peptides with ACE were mainly located in the binding pockets. For example, S2 pocket residues H353 and H513 were the top energy contributors for WWW/ACE and WWP/ACE complexes, respectively. S1 pocket residue Y523 contributed mostly to ACE’s binding with WYW, WQW, and WQP. Conversely, residues in binding pockets played a relatively less important role in the binding with C‐terminal‐C‐peptides. The average energy contribution of the binding pockets residues to WWC/ACE, WYC/ACE, and WQC/ACE complexes was −0.329, −0.045, and −0.430 Kcal/mol, respectively, which was lower than their energy contribution to the peptides with C‐terminal P and W (−0.777 Kcal/mol for WWW, −0.850 Kcal/mol for WWP, −0.507 Kcal/mol for WYW, −0.647 Kcal/mol for WYP, −1.244 Kcal/mol for WQW, and −1.026 Kcal/mol for WQP). The weak binding affinity between pocket residues and peptides with C‐terminal C may be a main reason for the complex's low stability. Interestingly, binding free energy of WWW/ACE complex was lower than that of WWP/ACE complex; however, the total energy of the pocket residues to WWW/ACE complex was higher than their energy to WWP/ACE complex. Similar results were observed for WYW/ACE and WYP/ACE complexes. Therefore, amino acids positioned outside the binding pockets played an essential role for ACE’s binding with C‐terminal W peptides. After comparison of the amino acids positioned outside the binding pockets, it was found that S355, V380, and V518 held more energy contribution than other nonbinding pocket residues. The positions of the three amino acids versus the binding pockets were illustrated in Figure 5a. It was assumed that ACE’s classical binding pockets may not be completely applicable to tripeptides when ACE and tripeptide were combined. We supposed S355, V380, and V518 should be included in the binding pockets, and the structure of the newly proposed ACE binding pockets was illustrated in Figure 5b and 5c. Furthermore, the interactions between WWW and the three key residues were shown in Figure 5d. S355 formed a H‐bond with WWW, which contributed to the stability of the complex, whereas hydrophobic interactions between WWW and S518 and V380 increase its binding affinity. When designing and modifying ACE inhibitory tripeptides for better inhibition effects, S355, V380, and V518 should also be regarded as key elements.

**TABLE 2:** Top energy contribution residues of ACE for the binding with C‐terminal W and P peptides

WWW/ACE		WQW/ACE		WYW/ACE		WWP/ACE		WYP/ACE		WCP/ACE	
Residue	Energy	Residue	Energy	Residue	Energy	Residue	Energy	Residue	Energy	Residue	Energy
H353	−6.682	Y523	−5.702	Y523	−6.794	H513	−5.584	R522	−4.912	Y523	−5.336
E411	−6.25	S355	−5.326	H383	−5.786	Y523	−5.234	W357	−4.636	A354	−3.552
A354	−5.97	H383	−4.728	E411	−5.786	H383	−3.888	S355	−4.568	H513	−2.942
V380	−4.064	E384	−4.158	E384	−4.14	V380	−3.334	P519	−4.496	H353	−2.852
H383	−4.058	H513	−4.04	V380	−3.57	A354	−3.074	V518	−3.512	Y520	−2.772
S355	−1.722	Y520	−4.04	H387	−2.388	H353	−2.834	Y523	−3.368	S355	−2.768
F391	−1.718	E411	−4.01	Y520	−2.026	K511	−2.404	A354	−3.032	H383	−1.688
W357	−1.674	V380	−3.316	F527	−1.808	E411	−2.056	H513	−2.502	K511	−1.468
H410	−1.488	H387	−2.362	H353	−1.236	S355	−2.016	A356	−1.674	V518	−1.326
H387	−1.398	F527	−1.764	H410	−1.194	E384	−1.608	H353	−1.516	V380	−1.27
V518	−1.31	H353	−1.682	F457	−0.998	V518	−1.416	Y520	−1.17	H387	−1.236
P163	−1.254	K511	−1.54	A354	−0.94	F457	−1.234	N66	−0.994	A356	−1.136
V351	−0.87	F457	−1.538	F391	−0.894	H387	−1.232	F512	−0.824	F457	−1.052
F512	−0.778	A356	−1.474	V379	−0.61	F512	−1.108	H383	−0.328	F512	−1.014
H513	−0.658	W357	−1.296	H513	−0.602	F527	−0.968	V351	−0.308	E411	−0.848

Note

The unit of energy is Kcal· mol^‐1^.

## 5 CONCLUSION

In conclusion, experimentation and theoretical calculation methods were combined in this work to elucidate the characteristics and mechanisms of ACE inhibitory peptide by a thorough analysis of all 8,000 tripeptides. The IC_50_ values of the five top‐rated peptides ranged from 5.86 to 21.84 μM, which showed that they were potential alternatives of ACE drugs. By structure–activity analysis, it was found that aromatic amino acid positioned at whether C‐ or N‐terminus of a tripeptide was linked to high ACE inhibitory activity. This characteristic of ACE inhibitor could guide the processing of antihypertensive functional foods. Additionally, electrostatic energy between ACE and its high affinity tripeptides was more requisite in keeping the stability of the complex than the other energies. S355, V380, and V518, three residues positioned around the classical binding pockets of ACE, also played a key role in ACE's binding. Therefore, for tripeptides, their binding pockets in ACE were redefined. The energy analysis allows a better understanding of the binding mechanisms of the ACE inhibitory peptides, which was useful in the design of ACE inhibitors. However, one limitation of our work was that whether the top‐ranked peptides are C‐domain‐selective ACE inhibitors was not verified. It has been reported that ACE inhibitors with C‐domain specificity have the potential for blood pressure control with fewer adverse effects (Acharya et al., 2003). Therefore, our future work will focus on the screening for ACE inhibitory peptides with C‐domain specificity, as well as the analysis of their characteristics.

## CONFLICTS OF INTEREST

The authors confirm that they have no conflicts of interest with respect to the work described in this manuscript.

## ETHICAL APPROVAL

This study does not involve any animal or human testing.

## Supporting information

Table S1
