# Corrigendum

**DOI:** 10.1002/fsn3.2497

**Published:** 2021-07-28

In the article by Chen et al., 2021. There is an error in the affiliation of the last and corresponding author, Lujia Zhang. The affiliation should read as:

1 State Key Laboratory of Bioreactor Engineering, School of Biotechnology, East China University of Science and Technology, Shanghai, China

2 Shanghai Engineering Research Center of Molecular Therapeutics and New Drug Development, School of Chemistry and Molecular Engineering, East China Normal University, Shanghai, China

3 NYU‐ECNU Center for Computational Chemistry at NYU Shanghai, Shanghai, China
